# The effect of SNPs in CYP450 in chloroquine/primaquine *Plasmodium vivax* malaria treatment

**DOI:** 10.2217/pgs-2016-0131

**Published:** 2016-10-21

**Authors:** Vinicius A Sortica, Juliana D Lindenau, Maristela G Cunha, Maria DO Ohnishi, Ana Maria R Ventura, Ândrea KC Ribeiro-dos-Santos, Sidney EB Santos, Luciano SP Guimarães, Mara H Hutz

**Affiliations:** 1 Departamento de Genética, Universidade Federal do Rio Grande do Sul, Porto Alegre, RS, Brazil; 2 Laboratório de Microbiologia e Imunologia, Universidade Federal do Pará, Belém, PA, Brazil; 3 Programa de Ensaios Clínicos em Malária, Instituto Evandro Chagas, Sistema de Vigilância Sanitária, Ministério da Saúde, Ananindeua, PA, Brazil; 4 Laboratório de Genética Humana e Médica, Universidade Federal do Pará, Belém, PA, Brazil; 5 Unidade de Bioestatística, Grupo de Pesquisa e Pós Graduação, Hospital de Clínicas de Porto Alegre, Porto Alegre, RS, Brazil

**Keywords:** chloroquine, *CYP2C8*, *CYP2C9*, *CYP3A5*, malaria, pharmacogenomics, *Plasmodium vivax*, primaquine, treatment

## Abstract

**Background:**

Chloroquine/primaquine is the current therapy to eliminate *Plasmodium vivax* infection in the Amazon region.

**Aims:**

This study investigates *CYP1A2*, *CYP2C8*, *CYP2C9*, *CYP3A4* and *CYP3A5* genetic polymorphisms influence on cloroquine/primaquine treatment.

**Patients & methods:**

Generalized estimating equations analyses were performed to determine the genetic influence in parasitemia and/or gametocytemia clearance over treatment time in 164 patients.

**Results:**

An effect of *CYP2C8* low-activity alleles on treatment was observed (p = 0.01). From baseline to first day of treatment, wild-type individuals achieved greater reduction of gametocytes than low-activity allele carriers. *CYP2C9* and *CYP3A5* genes showed a trend for gametocytemia and parasitemia clearance rates.

**Conclusion:**

Future studies should be performed to access the extent of *CYP2C8*, *CYP2C9* and *CYP3A5* gene polymorphisms influence on cloroquine/primaquine treatment.

First draft submitted: 27 July 2016; Accepted for publication: 25 August 2016; Published online: 21 October 2016

**Figure 1.**
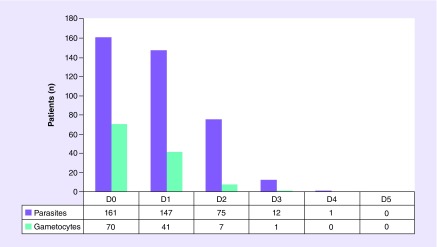
**Number of patients with parasites and gametocytes in blood during the days of chloroquine/primaquine treatment.**

**Figure 2.**
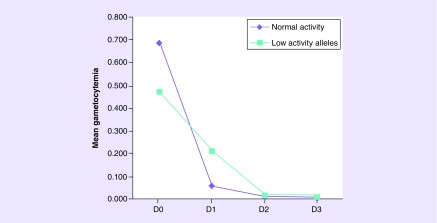
**Mean gametocytemia level reduction during chloroquine/primaquine regimen according to *CYP2C8* phenotypes.** Generalized Estimating Equations method with age, gender, co-medication, gametocytemia baseline level and genetic ancestry as co-variates; p_*Bonferroni*_ = 0.01 and *d* = 0.44.

**Figure 3.**
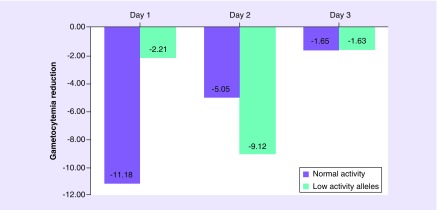
**Effect in mean gametocytemia reduction from baseline during choloroquine/primaquine regimen comparing *CYP2C8* phenotypes.** Generalized Estimating Equations method. Day 1, p = 0.007; day 2, p = 0.15; and day 3, p = 0.10.

## Background

Individual variation in drug disposition and response make effective drug prescribing a clinical challenge. Differences in drug response make the usual dosage regimen therapeutically effective in most patients, but some individuals do not experience any beneficial effect or suffer from drug toxicity. Genetic polymorphisms are major factors in Phase I and II metabolizing enzymes that influence pharmacokinetics in drug response [1]. Tropical diseases usually need multiple drug therapies to control infections that make pharmacogenetic studies in such diseases still more complex.

*Plasmodium vivax* is the major cause of malaria disease outside Africa, and it is an important morbidity and mortality factor in the Amazonian region [2]. The WHO recommended chloroquine (CQ) and primaquine (PQ) combined therapy as first choice treatment protocol for uncomplicated *P. vivax* malaria in CQ susceptible areas [2,3]. CQ is a 4-aminoquinoline derivative of quinine, and has been the most widely used antimalarial drug since 1946. This drug has effects as schizontocide and gametocide, and is metabolized by CYP450 isozymes 2C8, 3A4, 3A5 and, to a lesser extent, 2D6 [4–6]. The 8-aminoquinoline PQ, a quinine derivative, is an important gametocytocide and also is the unique effective drug against *P. vivax* and *Plasmodium ovale* hypnozoites. PQ is metabolized by CYP1A2, CYP3A4, CYP2D6 and CYPC19 isoforms in different extent to form diverse metabolites [7–10]. This drug could lead to severe hemolytic anemia in subjects with glucose-6-phosphate dehydrogenase (G6PD) deficiency and this condition needs to be investigated before PQ prescription in some populations [11].

Interindividual variability in CQ and PQ concentrations and effect was reported in Africa and Asia, which may affect treatment outcome in these populations [12–17]. *P. vivax* resistance to CQ, treatment noncompliance, medication suboptimum dose, patient health and/or nutritional status, drug–drug interactions are some factors that could lead to treatment failure [18]. However, genetic polymorphisms in CQ and PQ metabolizing enzymes that might influence drug availability and response to malaria therapeutic regimen were never investigated; therefore, the present study aims to evaluate whether genetic polymorphisms in *G6PD*, *CYP1A2*, *CYP2C8*, *CP2C9*, *CP3A4* and *CYP3A5* influence *P. vivax* malaria treatment response.

## Patients & methods

### Study population

The study cohort consisted of 164 *P. vivax* malaria patients followed during malaria treatment period from 2007 to 2009. All subjects were born in Pará state in the Brazilian Amazonian region, which presents different risk of infection and transmission among distinct regions and cities [19]. Patients were diagnosed and treated in Belém, Pará state at the Evandro Chagas Institute. Patients were aged between 12 and 88 years (36.0 ± 15.6 years). Twenty-nine patients (17.6%) use other medications in combination to CQ and PQ to treat malaria symptoms or pre-existing diseases. Sample collection and ancestry determination were previously described [20]. Patients were clinically examined and received the standard 1500 mg of CQ associated with 210 mg of PQ treatment in a short regimen as recommended by the Brazilian health authorities [21]. The therapeutic regimen was administered as CQ 600 mg and PQ 30 mg in the first day, followed by CQ 450 mg and PQ 30 mg in the second and third days, and PQ 30 mg in the last 4 days to all patients included in the study. This schedule was used because according to the Brazilian health authorities a shorter treatment time facilitates treatment adhesion in isolated Amazonian regions.

Treatment response was daily accompanied by clinical examinations. *P. vivax* asexual and sexual (gametocyte) forms density per μl of blood was daily estimated by counting the number of parasites per 100 fields and double-checked blindly by two expert microscopists as recommended by Brazilian ministry of health [22]. Patients were followed at the Evandro Chagas Institute for 6 months to identify relapse episodes. This follow-up time is the expected period in which relapses are expected to occur in the Amazonian region [23]. Patients were only considered with relapses if they presented malaria symptoms again and reside in urban areas with no risk of malaria transmission and did not travel to endemic areas. The incidence of malarial infections in the Brazilian Amazonian region was estimated as 6.3/1000 inhabitants. However, the transmission rate is variable in different localities. Usually it is higher in gold-digging areas and lower or absent in urban areas [19].

All subjects provided their written informed consent to participate in this study. The Ethics Committees of the Evandro Chagas Institute and Federal University of Pará approved the study protocol.

### Genotyping

Genomic DNA from all patients was extracted from subject's peripheral blood leukocytes using proteinase K digestion and standard phenol–chloroform procedures [24]. Reactions were performed in a total of 8 μl containing 10 ng of genomic DNA. The 13 SNPs in CYP450 and *G6PD* genes were determined by allelic discrimination assays (7500 Real Time PCR System^®^, Applied Biosystems, CA, USA) using Taqman 5′-nuclease assays^®^ (Applied Biosystems) (Table 1), according to the manufacturer's recommended protocol.

### Statistical analysis

Allele and genotype frequencies were estimated by gene counting, and haplotype frequencies and linkage disequilibrium were estimated with PHASE 2.1.1 [25]. Deviation from Hardy–Weinberg equilibrium was assessed by Qui-square tests with Bonferroni correction. The individual proportions of European, African and Amerindian genetic ancestry were estimated using the STRUCTURE software 2.3.3 [26,27]. Analyses of the effect of different genotypes on the efficacy of the treatment were performed using a generalized estimating equation (GEE) to determine the genetic influence in parasitemia or gametocytemia clearance over time. GEE is a repeated measure analysis focused on average changes in response over time and the impact of covariates on these changes. This method models the mean response as a linear function of covariates of interest via a transformation or link function and can be used in studies in which data are asymmetric or the distribution of data is difficult to verify due to small sample size [28]. GEE was performed considering a Gaussian distribution with an identity link function and an exchangeable correlation matrix structure in SPSS18.0 (IBM company) statistical package for Windows^®^ (IL, USA). Parasitemia and gametocytemia levels were log-transformed before analysis because of their asymmetric distribution, but untransformed data are shown in ‘Figures’ and ‘Tables’. Age, gender, co-medication, parasitemia baseline level, gametocytemia baseline level and genetic ancestry entered in models as covariates based on conceptual analyses of the literature and/or by means of a statistical definition (association with the study factor and with the outcome at p ≤ 0.15). Bonferroni correction for multiple comparisons was performed and corrected p-values were presented. Cohen's *d*-test was calculated to determine the effect sizes based on standardized differences between the means, that is, the difference between the means of the two conditions in terms of standard (z) scores [29]. Statistical significance was defined as a two-tailed p < 0.05.

## Results

After 7 days of treatment all patients presented negative results for parasites and gametocytes in blood. Parasitemia levels were reduced to 0 after 5 days of treatment and gametocytes were reduced to 0 after 4 days of treatment (Figure 1). No patient abandoned treatment and adverse drug reactions were not reported. After treatment, 27 patients (16.5%) presented relapses and repeated the therapeutic regimen.

### G6PD genotypes & phenotypes

Based on *G6PD* 202G>A and 376A>G SNPs only three malaria patients showed Gd A^-^ deficiency and four women were 202A and 376G carriers. Allele and genotype frequencies for *G6PD* SNPs are presented in Table 2. Patients with G6PD deficiency did not present adverse reactions to CQ/PQ treatment; therefore, *G6PD* genotypes were not considered as a confounder variable in this population study.

### Influence of CYP in parasitemia & gametocytemia clearance

*CYP1A2*, *CYP2C8*, *CYP2C9*, *CYP3A4* and *CYP3A5* allele frequencies in the investigated sample are shown in Table 3. The genotype distribution did not deviate significantly from Hardy–Weinberg equilibrium. A functional approach was used to group genotypes. Therefore, *CYP2C8* reduced activity allele carriers were compared with subjects with wild type alleles to explore the effect of these genes on outcomes. After adjustment for age, gender, co-medication, parasitemia baseline level, gametocytemia baseline level, and genetic ancestry in the GEE analysis, only *CYP2C8*, was associated with gametocytemia clearance rates.

*CYP2C8*-reduced activity variants (*2, *3, *4) are low-activity alleles. Demographic and clinical characteristics of the patients according to metabolism status are shown in Table 4. Figure 2 shows the trajectory of gametocyte elimination based on findings from the GEE model, including treatment over time and the presence of low-activity alleles as main effects, age, gender, co-medication, gametocytemia baseline level and genetic ancestry as covariates (conceptual confounders), and significant interactions between these factors during treatment. A significant effect of CYP2C8*2/*3/*4 alleles (p_*Bonferroni*_ = 0.01) on treatment was observed and a significant interaction effect between low-activity alleles and treatment over time (p = 0.017) was also observed although after Bonferroni correction it was no longer significant. From baseline to the first day of treatment, homozygous individuals for wild-type *CYP2C8* achieved greater reduction (p = 0.007) of gametocytes than individuals without this genotype (Figures 2 & 3). The *CYP2C8* polymorphism estimated effect size (0.44) determines a moderate clinical effect considering Cohen's suggestion [30].

*CYP2C9* gene was associated with gametocytemia clearance rates (p = 0.037), but this association was no longer significant after Bonferroni correction (Table 5). No main effect was observed for *CYP3A5*, but an interaction between gene over time on parasitemia elimination rate during treatment was disclosed (p = 0.007) (Table 5). CYP3A5*3 and *6 carriers showed a lower rate of parasite elimination rate during treatment compared with wild-type carriers. After Bonferroni correction only a trend for these associations was observed, and they were not further explored.

## Discussion

Metabolism plays an important role in drug disposition with pharmacological and toxicological implications in the use of therapeutic drugs. CYPs are expressed mostly in the liver representing the most important Phase I drug-metabolizing enzymes that oxidize several endogenous substances and xenobiotics, as most medications [31].

The human *CYP2C8* and *CYP2C9* genes are mapped to chromosome 10q24 and exhibit similar substrate specificity but with distinct metabolizing rates. CYP2C8 is mainly expressed in the liver and metabolizes near 5% of drugs cleared by Phase I reactions, while CYP2C9, that is also an abundant enzyme expressed in the liver, metabolizes approximately 15% of clinical drugs [32–35]. In the present study, *CYP2C8*-reduced activity alleles carriers showed lower rates of gametocyte elimination as compared with homozygous wild-type allele *1A. CYP2C8*4 is a missense mutation, which promotes a lower enzyme activity *in vitro* than the wild-type allele *1A, and similarly CYP2C8*2 and CYP2C8*3 also present a markedly decrease activity *in vitro* [36,37]. In Africa, CYP2C8*2 and CYP2C8*3 were associated with impaired metabolism of the antimalarial amodiaquine while CYP2C8*4 was not identified [38]. *CYP2C9*-reduced activity allele carriers also showed a lower gametocytemia clearance rate during treatment period, although not significant after Bonferroni correction. CYP2C9 was not related to CQ or PQ metabolism, however, linkage disequilibrium between *CYP2C9* and *CYP2C8* alleles was already reported in the admixed Brazilian population where those genes constitute a haplotypic block [39]. Linkage disequilibrium or an overlap of these enzyme functions are possible explanations for the trend observed in CQ/PQ treatment outcome. The lower rate of gametocyte elimination by CYP2C8*2/*3/*4 allele carriers observed herein indicates a slow response to treatment. CQ has a major effect as schizontocides in erythrocytes, but is also effective against *P. vivax* gametocytes. PQ has major effect as gametocytocide and hypnozoitocide in liver and is not metabolized by those CYPs isoenzymes. Besides *CYP2C8* alleles were associated with slow gametocytemia clearance in CQ/PQ-associated regimen, the effect of PQ probably was sufficient to reach an adequate gametocyte clearance for all patients after 4 days of treatment. The synergistic effect of both drugs could prevent an ineffective response to treatment.

The present study also reported a trend for a gene over time interaction between lower parasite elimination rates during malaria treatment and *CYP3A5* splicing defect alleles (CYP3A5*3 and *6) carriers. Taken together, these results indicate that *CYP2C8*, *CYP2C9* and *CYP3A5* genetic variants potentially influence in CQ/PQ malaria treatment and should be better evaluated further in larger studies to prevent ineffective treatment and adverse effects.

Antimalarial drugs were usually administered in combination therapies making difficult pharmacogenetics and pharmacokinetics data interpretation. The present study was performed with patients in normal treatment conditions and differences in age, gender, genetic ancestry and use of other drug together with malaria treatment were taken into account in the analyses. Multiple comparison correction tests and effect size estimates were also performed to address reliable results. Nevertheless, the study has some limitations: it was not possible to infer or control the interindividual immune response variability in malaria patients, which contributes to malaria treatment response; it was not possible to determine if patients were *P. vivax*-infected with CQ or PQ-resistant strains; however, the patients did not present relapses before 28 days, which is considered a CQ resistance *in vivo* test [40]; CQ and PQ plasma concentrations were not assessed and CYP genetic variance influence on pharmacokinetics could not be directly correlated; the study design does not allow the investigation of the genetic influence on malaria relapses. Besides these limitations, the present results and their effect size reported reinforce the potential role of pharmacogenomics in *P. vivax* malaria treatment.

## Conclusion

The present study reported for the first time the influence of *CYP2C8* gene on gametocyte clearance rate on patients under chloroquine/primaquine malaria treatment. The study also indicates a possible role of *CYP2C9* and *CYP3A5* in malaria treatment. Future studies with larger sample sizes are needed to clarify the extent of *CYP2C8*, *CYP2C9* and *CYP3A5* gene polymorphism influences on CQ/PQ treatment outcome.

## Future perspective

Even after all the efforts to develop a multiple drug therapy that has a good response to malaria treatment, this disease still is an important morbidity and mortality factor in several world regions, among them is the Amazonian region. For now, pharmacogenetic studies of this kind of disease are scarce, mainly because of their complexity. However, the present study reports an important contribution to the development of personalized treatment for malaria.

**Table 1.** **List of SNPs genotyped in the present study.**

**Gene**	**SNP**	**dbSNP ID**	**Assay ID**
*CYP1A2*	-360G>A	rs2069514	C__15859191_30
	-163C>A	rs762551	C__8881221_40

*CYP2C8*	805A>T	rs11572103	C__30634034_10
	792C>G	rs1058930	C__25761568_20
	416G>A	rs11572080	C__25625794_10

*CYP2C9*	3608C>T	rs1799853	C__25625805_10
	1003C>T	rs28371685	C__30634132_70
	42614A>C	rs1057910	C__27104892_10
	1080C>G	rs28371686	C__27859817_40

*CYP3A4*	-392A>G	rs2740574	^†^

*CYP3A5*	14690G>A	rs10264272	C__30203950_10
	6986A>G	rs776746	C__26201809_30

*G6PD*	202G>A	rs1050828	C__2228686_20
	376A>G	rs1050829	C__2228694_20

^†^Custom assay.

dbSNP: A database of SNP.

**Table 2.** ***G6PD* allele and genotype frequencies.**

**SNP**	**Alleles, n (%)**			**Genotypes, n (%)**	
		**Male**	**Female**		
202G>A	G	105 (98.1)	89 (93.0)	GG	43 (89.6)
	A	2 (1.9)	7 (7.0)	GA	3 (6.3)
				AA	2 (4.2)

376A>G	A	94 (88.7)	86 (91.5)	AA	40 (85.1)

	G	12 (11.3)	8 (8.5)	AG	6 (12.8)
				GG	1 (2.1)

202A + 376G determine A^-^ phenotype.

**Table 3.** **CYP450 allelic frequencies.**

**Gene**	**Alleles**	**n**	**Frequency (%)**
*CYP1A2*	*1A	122	37.2
	*1C	127	38.7
	*1F	79	24.1

*CYP2C8*	*1A	283	86.3
	*2	19	5.8
	*3	21	6.4
	*4	5	1.5

*CYP2C9*	*1A	291	88.7
	*2	23	7.0
	*3	9	2.8
	*11	5	1.5

*CYP3A4*	*1A	272	82.9
	*1B	56	17.1

*CYP3A5*	*1A	80	24.4
	*3	236	71.9
	*6	12	3.7

**Table 4.** **CYP2C8 group phenotypes main characteristics.**

**Characteristics**	CYP2C8*1	CYP2C8*2/*3/*4 allele carriers	**p-value**
n^†^	118	45	

Age (years)	36.0 (15.6)	35.0 (15.1)	0.6^‡^

Gender; male (%)	68.1	71.1	0.4^§^

Baseline parasitemia (parasites/μl)	8374.3 (50–40,000)	9222.2 (100–75,000)	0.7^¶^

Baseline gametocytemia (gametocytes/μl)	126.5 (0–4500)	73.3 (0–500)	0.7^¶^

Genetic ancestry:			
– African	0.239 (0.10)	0.254 (0.10)	0.2^¶^
– European	0.415 (0.10)	0.416 (0.13)	0.6^¶^
– Native American	0.345 (0.12)	0.329 (0.13)	0.5^¶^

Values for age and genetic ancestry are expressed as mean (standard deviation).

Values for parasitemia and gametocytemia are expressed as median (range).

^†^One individual was not included in this analysis due to genotyping failure.

^‡^Student's *t*-test.

^§^Fisher exact test.

^¶^Mann–Whitney test.

**Table 5.** **Parasitemia and gametocytemia reduction association with *CYP* genes and gene interaction with time.**

**Gene**	**Parasitemia**					**Gametocytemia**				
	**p-value**		**p_*Bonferroni*_**		***d*^†^**	**p-value**		**p_*Bonferroni*_**		***d*^†^**
	**Gene**	**Gene × time**	**Gene**	**Gene × time**		**Gene**	**Gene × time**	**Gene**	**Gene × time**	
*CYP1A2*	0.14	0.29	NS	NS		0.20	0.31	NS	NS	

*CYP2C9*	0.89	0.08	Ns	NS		0.037	0.21	0.37	NS	0.24

*CYP3A4*	0.79	0.37	NS	NS		0.93	0.90	NS	NS	

*CYP3A5*	0.80	0.007	NS	0.07	0.53	0.49	0.77	NS	NS	

^†^Effect size Cohen's *d*-test.

NS: Not significant.

**Executive summary**

- The WHO recommended as first choice treatment protocol for uncomplicated *Plasmodium vivax* malaria chloroquine (CQ) and primaquine (PQ) combined therapy, in CQ susceptible areas.
- CQ and PQ are metabolized by several CYP450 (CYP) isozymes. Therefore, genetic polymorphisms in these metabolizing enzymes might influence *P. vivax* malaria treatment response.
- A total of 164 *P. vivax* malaria patients followed during malaria treatment with CQ and PQ were genotyped for 13 SNPs in CYP450 and *G6PD* genes.
- Analyses of the effect of different genotypes on treatment efficacy were performed using generalized estimating equations to determine the genetic influence on parasitemia or gametocytemia clearance over time.
- From baseline to the first day of treatment, wild-type *CYP2C8* homozygous individuals achieved greater reduction of gametocytes than individuals without this genotype.
- *CYP2C9* gene was associated with gametocytemia clearance rates and CYP3A5*3 and *6 carriers showed a lower rate of parasite elimination rate during treatment compared with wild-type carriers. However, only a trend for these associations was observed after Bonferroni correction.
- Taken together, these results indicate that *CYP2C8*, *CYP2C9*, *CYP3A5* genetic variants potentially influence CQ/PQ malaria treatment and should be better evaluated in larger studies to prevent ineffective treatment and adverse effects.
